# Keep on Knocking

**Published:** 1913-12

**Authors:** 


### Keep on Knocking

One of our subscribers recently said to another, who told us: "That blamed journal takes two hours of my time now. I have to read it from cover to cover." Don't forget the advertising pages and don't forget to mention this or any other journal in writing to advertisers. This policy and the W. P. B. for circulars will give you better literature.

